# *Graptopetalum paraguayense* Ameliorates Airway Inflammation and Allergy in Ovalbumin- (OVA-) Sensitized BALB/C Mice by Inhibiting Th2 Signal

**DOI:** 10.1155/2013/237096

**Published:** 2013-06-17

**Authors:** Bao-Hong Lee, Yu-Hsiang Cheng, She-Ching Wu

**Affiliations:** ^1^Department of Food Science, National Chiayi University, Taiwan; ^2^Department of Science, University of Auckland, New Zealand

## Abstract

Role of inflammation-induced oxidative stress in the pathogenesis and progression of chronic inflammatory airways diseases has received increasing attention in recent years. Nuclear factor erythroid 2-related factor 2 is the primary transcription factor that regulates the expression of antioxidant and detoxifying enzymes. *Graptopetalum paraguayense* E. Walther, a vegetable consumed in Taiwan, has been used in folk medicine for protection against liver injury through elevating antioxidation. Recently, we found that gallic acid is an active compound of *Graptopetalum paraguayense* E. Walther, which has been reported to inhibit T-helper 2 cytokines. Currently, we assumed that *Graptopetalum paraguayense* E. Walther may potentially protect against ovalbumin-induced allergy and airway inflammation. Results demonstrated that *Graptopetalum paraguayense* E. Walther ethanolic extracts (GPE) clearly inhibited airway inflammation, mucus cell hyperplasia, and eosinophilia in OVA-challenged mice. Additionally, GPE also prevented T-cell infiltration and Th2 cytokines, including interleukin- (IL-)4, IL-5, and IL-13 generations in bronchial alveolar lavage fluid. The adhesion molecules ICAM-1 and VCAM-1 were substantially reduced by GPE treatment mediated by Nrf2 activation. Moreover, GPE attenuated GATA3 expression and inhibited Th2 signals of the T cells. These findings suggested that GPE ameliorated the development of airway inflammation through immune regulation.

## 1. Introduction 

Asthma is recognized as a common pulmonary disease and is a serious public health problem worldwide. In the last two decades, asthma-related morbidity and mortality have increased [1]. Airway hyperreactivity (AHR), chronic pulmonary inflammation with eosinophil infiltration in the lungs, and mucus hypersecretion in the airways are hallmarks of allergic asthma. Reactive oxygen species (ROS) are generated in the airway epithelial cells in response to a variety of stimuli. In addition to releasing inflammatory cytokines and chemokines, airway epithelial cells express adhesion molecules on their cell surface. The production of ROS causes airway inflammation, which involves narrowing of airways, thickening of the epithelium, and secretion of large amounts of mucus, as well as infiltration of eosinophils, T cells, and other inflammatory cells [2–4].

Both eosinophils and T-helper (Th) 2 lymphocytes play pathogenic roles in asthma [5]. Eosinophils are commonly associated with allergic inflammation and act as effector cells in the pathogenesis of this disease by releasing cytotoxic granule proteins [6]. Eotaxin is a potent chemoattractant for eosinophils, and its levels are generally elevated after asthma induction. An imbalance between Th1 and Th2 leads to the development of an allergic disease. The levels of Th2 cytokines, including IL-4, IL-5, and IL-13, typically increase during an allergic disease. These cytokines have important roles in airway infiltration, eosinophil activation, induction of immunoglobulin E (IgE) production, mucus secretion, and release of a variety of inflammatory mediators [7].

The transcription factor nuclear factor erythroid 2-related factor 2 (Nrf-2) is involved in lung defense against oxidative injury. After translocation to the nucleus, Nrf-2 binds to antioxidant response elements (AREs) located in promoter regions of relevant genes and induces the transcription of numerous antioxidant and cellular defense genes, including glutathione S-transferase (GST), NADP(H):quinone oxidoreductase 1 (NQO1), heme oxygenase-1 (HO-1), superoxide dismutase (SOD), catalase (CAT), and glutathione peroxidase (GPx) [8]. The roles of these enzymes and proteins in oxidative tissue stress have been widely discussed in the literature [9, 10]. Disruption of the Nrf-2 gene leads to severe allergen-driven airway inflammation and hyperresponsiveness in mice and increased expression of the T-helper type 2 (Th2) cytokines interleukin- (IL-) 4 and IL-13 in bronchoalveolar lavage fluid (BALF) and splenocytes after allergen challenge [11]. Nrf-2 also protects the lungs against the development of pulmonary fibrosis by regulating the cellular redox level and lung Th1/Th2 balance [12].

*Graptopetalum paraguayense* E., a vegetable consumed in Taiwan, has been used in folk medicine for protection against liver injury and hepatic fibrosis [13, 14]. In our recent study, we found that gallic acid is a major antioxidant of *Graptopetalum paraguayense* E. for hepatoprotection [15]. Moreover, we have reported that the level of gallic acid in *Graptopetalum paraguayense* E. was elevated via *Lactobacillus* fermentation [16]. Gallic acid has been confirmed to inhibit histamine release, proinflammatory cytokines in mast cells [17], and Th2 cytokines productions in T cells [18]. In addition, gallic acid has also been found in Goishi tea and has been reported to inhibit AHR in vivo [19]. These lines of evidence suggest that *Graptopetalum paraguayense* E. may have potential for asthma therapy; however, the actual efficacy remains uncertain. Therefore, we investigated the protective effects of *Graptopetalum paraguayense* E. against ovalbumin- (OVA-) induced asthma in vivo through Nrf2 activation in this study.

## 2. Methods

### 2.1. Materials

Aluminum hydroxide, ovalbumin (OVA), and gallic acid were purchased from Sigma Chemical Co. (St. Louis, MO, USA). Sodium dodecyl sulfate (SDS) was purchased from Merck (Darmstadt, Germany). The Bio-Rad protein assay dye was from Bio-Rad Laboratories (Hercules, CA, USA). OVA-specific immunoglobulin E (IgE) ELISA kit was purchased from BD Biosciences (San Diego, CA, USA).

### 2.2. Preparation of Extracts

The fresh *Graptopetalum paraguayense* E. Walther leaves, supplied from Wei-Shen farm (Chiayi, Taiwan), were washed, freeze-dried, and then ground to a powder. The *Graptopetalum paraguayense* E. extracts (GPE) were prepared by 95% ethanol extraction for 48 h, and the extraction solution was freeze-dried and stored at 4°C [15].

### 2.3. Murine Model of OVA-Induced Allergic Airway Inflammation

**Figure 1:**
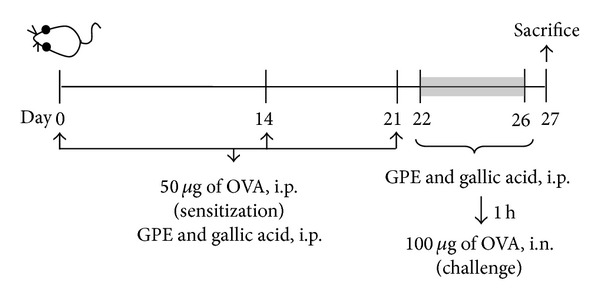
The flowchart of OVA-induced allergy and airway inflammation.

The experiment was carried out with female 6- to 8-week-old BALB/c mice obtained from National Laboratory Animal Center (Taipei, Taiwan). Mice were subjected to 12 h light/dark cycle with a maintained relative humidity of 60% and a temperature at 25°C (protocol complied with guidelines described in the “Animal Protection Law,” amended on Jan. 17, 2001 Hua-Zong-(1)-Yi-Tzi-9000007530, Council of Agriculture, Executive Yuan, Taiwan). Groups of 12 animals were used in all tests. Mice were sensitized on days 0, 14, and 21 by intraperitoneal injection with 50 *μ*g of OVA in 200 *μ*L PBS mixed with 1 mg aluminum hydroxide as an adjuvant [20]. On days 22 to 26, mice were challenged by intranasal inhalations of OVA (100 *μ*g) twice a day. Mice (*n* = 12) were sacrificed at 24 h after the last challenge (Figure 1). GPE (50 mg kg^−1^ bw and 200 mg kg^−1^ bw) and gallic acid (10 mg kg^−1^ bw) were administrated by intraperitoneal injection once a day while intraperitoneal induction and challenge with OVA. The gallic acid dosage (10 mg kg^−1^ bw) was equivalent to GPE (200 mg kg^−1^ bw) [15].

### 2.4. Collection of Bronchoalveolar Lavage (BALF)

The tracheas of mice were lavaged with two 0.8 mL aliquots of cold PBS. The collected fluid was called BALF, and absolute cell count for each cell type was calculated after determining the total cell count and differential cell count.

### 2.5. Lung Histopathology

At 24 h after the last OVA challenge, mice were sacrificed and the lungs were fixed in 10% neutral buffered formalin. After fixation, 5 mm paraffin sections were stained with hematoxylin and eosin (H&E) for the evaluation of inflammatory cell infiltration or with periodic acid-Schiff (PAS) for the identification of goblet cells. The slides were examined by a pathologist. The inflammatory score was graded on the following scale: 0, none; 1, minimal; 2, mild; 3, moderate; 4, severe. The number of PAS cells permillimeter of base membrane was measured for quantitative analysis.

### 2.6. RNA Preparation and Real-Time PCR

Total RNA was isolated using TRIzol (Life Technologies) according to the manufacturer's instructions. cDNA from 3 *μ*g of RNA was generated using SuperScript III First-Strand Synthesis System for RT-PCR (Life Technologies) according to the manufacturer's instructions. The reverse transcription product was diluted in water, and a volume corresponding to 30 ng of original RNA was used for real-time PCR. Real-time PCR amplification and detection were performed using the SYBR Green qPCRSuperMix-uracil DNA glycosylase (UDG) with ROX (Life Technologies) in a fluorescence thermal cycler (Step One Real-Time PCR System, Life Technologies) according to the manufacturer's protocol. Gene expression was normalized using beta-actin as a reference gene. The primers are beta-actin sense, 5′-CTA AGG CCA ACC GTG AAA AG-3′, and antisense, 5′-AGC CTG GAT GGC TAC GTA CAT-3′; intercellular adhesion molecule-1 (ICAM-1) sense, 5′-CTG GCT GTC ACA GAA CAG GA-3′, and antisense, 5′-AAA GTA GGT GGG GAG GTG CT-3′; vascular cell adhesion molecule-1 (VCAM-1) sense, 5′-CCC AAG GAT CCA GAG ATT CA-3′, and antisense, 5′-TAA GGT GAG GGT GGC ATT TC-3′; GST sense, 5′-AAG CCA GGA CTC TCA CTA-3′, and antisense, 5′-AAG GCA GTC TTG GCT TCT-3′; GCL sense, 5′-CGC CTG CGA AAA AAG TGC-3′, and antisense, 5′-TCA TTC AAG GTC TTT TGG ATA CAG TC-3′.

### 2.7. Flow Cytometry Analysis

BALF cell pellet was resuspended and stained with anti-CD4 antibody (Invitrogen, Carlsbad, CA, USA) and anti-CD8 antibody (BioLegend, San Diego, CA, USA) and analyzed by flow cytometry using a FACScan-LSR flow cytometer equipped with CellQuest software (BD Biosciences, San Jose, CA, USA).

### 2.8. Assay for Cytokines

Supernatant derived from the BALF was stored at −80°C until measurement. ELISA experiments were performed according to the manufacturer's instructions. The levels of interleukin- (IL-) 4, IL-5, IL-13, and interferon-gamma (IFN-*γ*) in BALF or serum were determined with ELISA kits from eBioscience (San Diego, CA, USA).

### 2.9. Thiobarbituric Acid Reactive Substances (TBARS) Assay

To determine lipid peroxidation, the TBARS assay was used. Lung tissue samples were crushed in RIPA lysis buffer (1% Triton X-100, 1% sodium deoxycholate, 5 mM iodoacetamide, 1% bovine hemoglobin, and 0.025% NaN3). The suspension was sonicated three times for 30 s and centrifuged at 3000 ×g for 10 min at 4°C. The 200 *μ*L of supernatant was added to 200 mL of ice-cold 10% trichloroacetic acid to precipitate proteins and incubated for 15 min on ice. The samples were centrifuged at 2200 ×g for 15 min. And the 200 *μ*L of supernatant was added to an equal volume of 0.67% thiobarbituric acid and incubated in a boiling water bath for 10 min. Samples were measured at 532 nm.

### 2.10. Immunoblot Analysis

After sacrificing, the blood of mice was collected and centrifugated (300 ×g for 15 min), and the peripheral blood mononuclear cells were obtained with Ficoll-Hypaque (1.077 g mL^−1^). Cells were lysed in ice-cold lysis buffer overnight and then centrifuged (12,000 ×g, 10 min) to recover the supernatant. The supernatant was taken as the cell extract. The samples were subjected to 10% SDS-polyacrylamide gel electrophoresis (PAGE). The protein spots were electrotransferred to a polyvinyledine difluoride (PVDF) membrane. The membrane was incubated with block buffer (PBS containing 0.05% of Tween 20 and 5% nonfat milk) for 1 h, washed with PBS containing 0.05% Tween 20 (PBST) three times, and then probed with 1 : 1000 diluted solution of primary antibody for overnight. The membrane was washed three times each for 5 min in PBST, shaken in a solution of HRP-linked secondary antibody, and washed three more times each for 5 min in PBST. The expressions of proteins were detected by enhanced chemiluminescent (ECL) reagent (Millipore, Billerica, MA, USA).

### 2.11. Statistical Analysis

The data were recorded as mean ± SEM. The statistical significance was determined by one-way analysis of variance (ANOVA) using the general linear model procedure of SPSS software (SPSS Institute, Inc.), followed by ANOVA with Duncan's test. Results were considered statistically significant at *P* < 0.05.

## 3. Results

### 3.1. Effect of GPE on Attenuating Inflammatory Cells Recruitment

**Figure 2:**
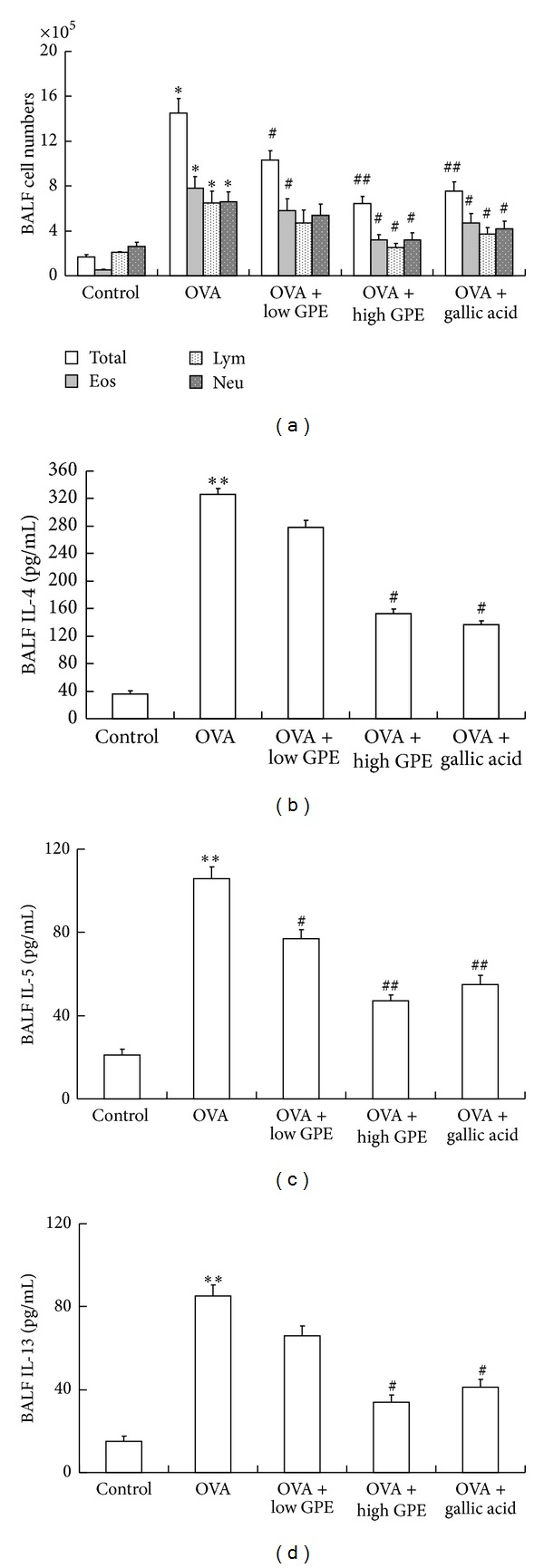
Attenuations of GPE in immune cell infiltration and Th2 cytokines (IL-3, IL-5, and IL-13) levels in BALF. Data were shown as mean ± SEM (*n* = 12). The left axis represents total cell and eosinophil populations, whereas the right axis represents lymphocyte and neutrophil populations. *Significantly different from control group at *P* < 0.05. ^#^Significantly different from OVA-challenged group at *P* < 0.05. **Significantly different from control group at *P* < 0.01. ^##^Significantly different from OVA-challenged group at *P* < 0.01. Eos, eosinophil; Lym, lymphocyte; Neu, neutrophil.

We examined the effects of GPE on the infiltration of inflammatory cells into the lung tissues of OVA-induced mice (Figure 1). BALF was collected after the last OVA challenge through intranasal instillation. Total and differential cell counts were performed. Results showed that the infiltration of inflammatory cells in the peribronchial and perivascular areas was induced by OVA, including increases in the number of total cells, eosinophils, lymphocytes, and neutrophils. However, GPE and gallic acid reduced inflammatory cell infiltration in these areas, as shown in Figure 2(a).

Numerous studies performed with animal models of pulmonary inflammation have shown that the levels of Th2 cytokines increase within the affected airways. Moreover, Th2 cells together with other inflammatory cells such as eosinophils play a major role in the initiation and pathological development of airway inflammation [21, 22]. As shown in Figures 2(b)–2(d), the levels of BALF Th2 cytokines, IL-4, IL-5, and IL-13, were all elevated by OVA induction and decreased by GPE and gallic acid treatments.

**Figure 3:**
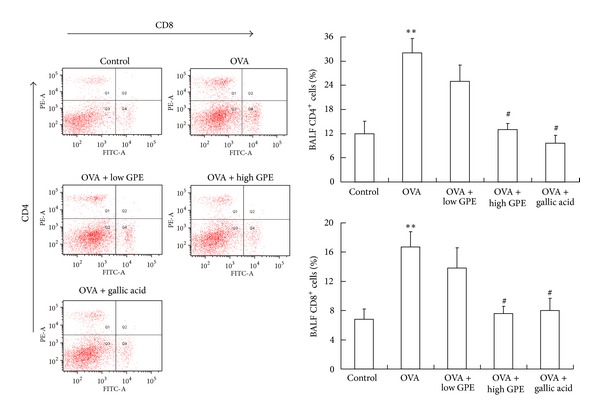
GPE-mediated inhibition of CD4 and CD8 T-cell infiltration into BALF. Analysis of T cells was performed by flow cytometry. Cells in the lower right quadrant represented CD8-positive cells and in the upper left quadrant represented CD4-positive cells. The percentages of CD4- and CD8-positive cells were densitometrically analyzed. Data were shown as mean ± SEM (*n* = 12). *Significantly different from control group at *P* < 0.05. ^#^Significantly different from OVA-challenged group at *P* < 0.05.

Therefore, we next examined T-cell levels in the BALF by flow cytometry. CD4 and CD8 T cells were clearly visible and abundant in OVA-challenged mice compared to control mice. Conversely, T-cell infiltration was diminished in OVA-challenged mice treated with GPE or gallic acid, compared to OVA-challenged mice, with the degree of infiltration being similar to that observed in the control mice (Figure 3).

**Figure 4:**
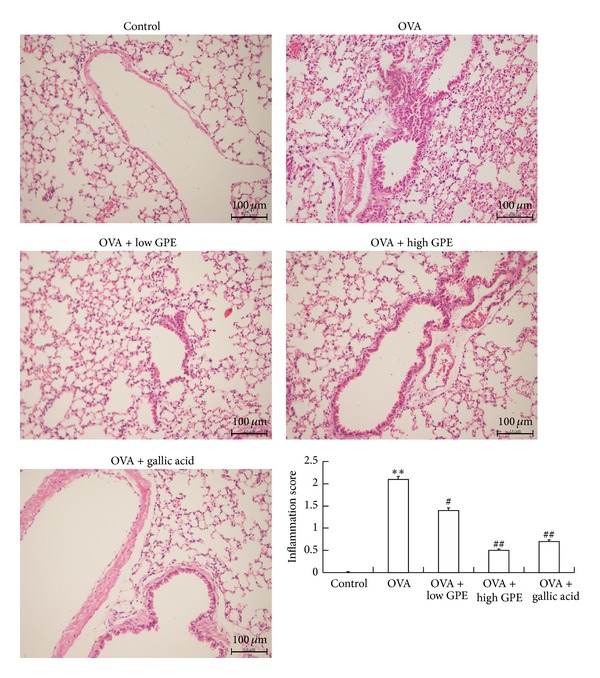
Reduction of GPE on infiltration of inflammatory cells into the lung tissues of OVA-challenged mice by HE stain. The inflammatory score was graded on the following scale: 0, none; 1, minimal; 2, mild; 3, moderate; 4, severe. Data were shown as mean ± SEM (*n* = 12). **Significantly different from control group at *P* < 0.01. ^#^Significantly different from OVA-challenged group at *P* < 0.05. ^##^Significantly different from OVA-challenged group at *P* < 0.01.

Histopathology analysis revealed inflammatory cell infiltration into the airways of OVA-challenged mice. However, HE staining revealed fewer inflammatory cell infiltrates in the OVA-treated mice receiving GPE and gallic acid than in the OVA-challenged mice (Figure 4).

### 3.2. Antimucus Activity of GPE and Gallic Acid

**Figure 5:**
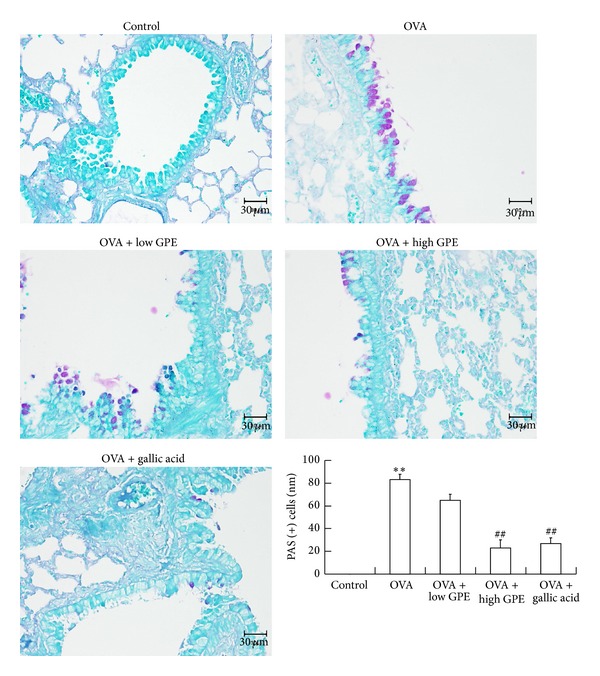
Suppression of GPE on hyperplasia of goblet cells into the lung tissues of OVA-challenged mice. Lung tissues were stained with PAS, and PAS-positive cells numbers per mm of base membrane were quantitatively analyzed. Data were shown as mean ± SEM (*n* = 12). **Significantly different from control group at *P* < 0.01. ^##^Significantly different from OVA-challenged group at *P* < 0.01.

Mucus hypersecretion is characteristic of airway inflammation. To assess whether GPE and gallic acid suppressed mucus overproduction as a result of goblet cell hyperplasia in the OVA-challenged mice, we stained lung sections with PAS. Bright violet staining indicative of mucus overproduction was clearly observed in the bronchial airways of the OVA-challenged mice compared to the PBS-treated control mice. In contrast, mucus staining was markedly diminished in the GPE- and gallic acid-treated mice compared to the OVA-challenged mice (Figure 5).

### 3.3. Inhibition of Oxidative Stress by GPE and Gallic Acid Mediated by Nrf2 Activation

**Figure 6:**
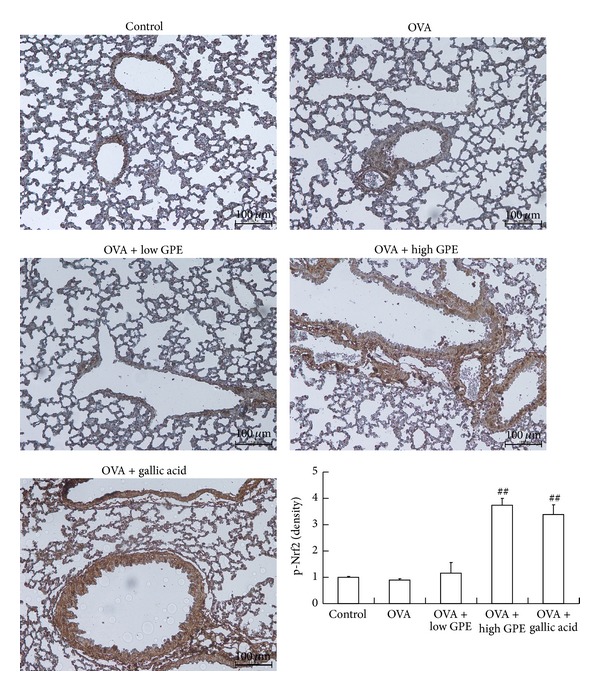
The p-Nrf2 (serine 40) was evaluated by IHC stain. Data were shown as mean ± SEM (*n* = 12). ^##^Significantly different from OVA-challenged group at *P* < 0.01.

**Figure 7:**
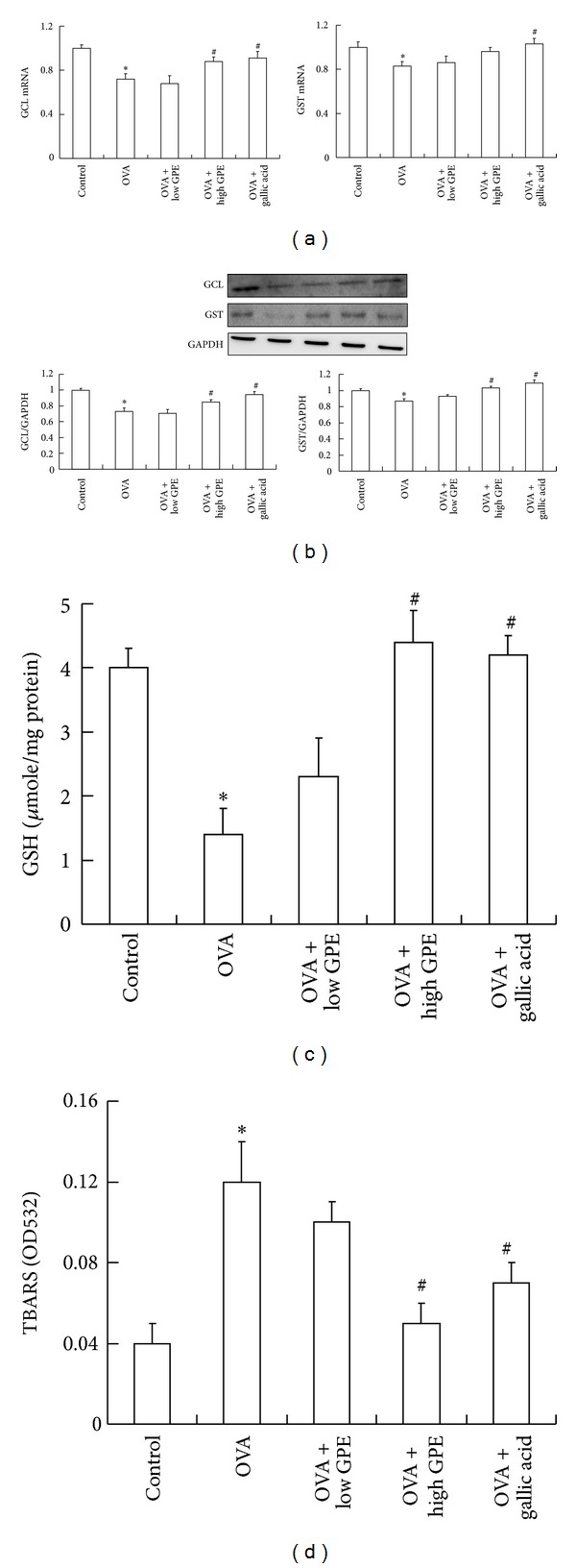
(a) The elevations of GPE on lung GCL and GST mRNA levels measured by real-time PCR. (b) The elevations of GPE on lung GCL and GST protein measured by western blot. (c) The upregulation of GPE on GSH level and (d) downregulation of GPE on lung lipid peroxidation in OVA-challenged mice. Data were shown as mean ± SEM (*n* = 12). *Significantly different from control group at *P* < 0.05. ^#^Significantly different from OVA-challenged group at *P* < 0.05.

Nrf2 is essential for ARE-mediated induction [9, 10]. Several studies have demonstrated that Nrf2 is activated by phosphorylation at serine 40 [23, 24]. IHC staining revealed that GPE and gallic acid markedly elevated the level of p-Nrf2 (serine 40) in the lungs of OVA-challenged mice (Figure 6). In addition, the mRNA and protein expression levels of molecules downstream of Nrf2, such as GCL and GST, were elevated in the lungs of the OVA-challenged mice (Figures 7(a) and 7(b)). The level of GSH was also increased (Figure 7(c)), suggesting that GPE may have potential for improving oxidative stress.

Given that increased oxidative stress can result in airway tissue damage via lipid peroxidation [20], we evaluated lipid peroxidation in lung homogenates. As shown in Figure 7(d), our results indicated that GPE and gallic acid significantly attenuated the generation of TBARS in the lungs owing to OVA induction.

### 3.4. Inhibitory Effects of GPE and Gallic Acid on VCAM-1 and ICAM-1

**Figure 8:**
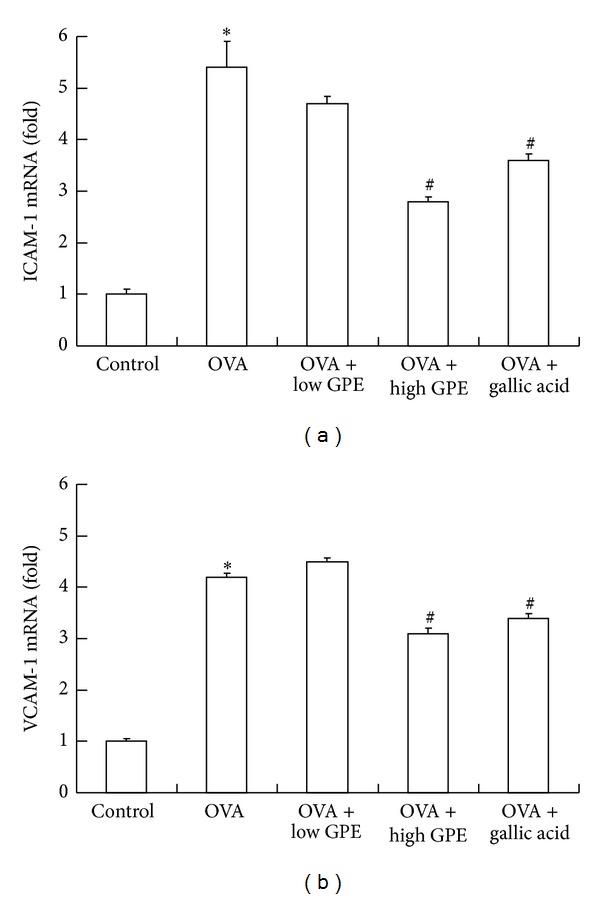
Attenuations of GPE on ICAM-1 and VCAM-1 mRNA of lung in OVA-challenged mice by real-time PCR analysis. Data were shown as mean ± SEM (*n* = 12). *Significantly different from control group at *P* < 0.05. ^#^Significantly different from OVA-challenged group at *P* < 0.05.

Leukocytes are recruited into tissues by adhesion molecules and chemokines. Adhesion molecule expression is induced in the endothelium or epithelium by several mediators, including cytokines or ROS, in a variety of chronic inflammatory conditions [25]. As GPE and gallic acid inhibited eosinophil, lymphocyte, and neutrophil recruitment into the lungs (Figure 2(a)), we sought to further elucidate the relationship between GPE and the inhibition of adhesion molecule production.  Figure 8 reveals that the mRNA levels of both ICAM-1 and VCAM-1 in the lungs of the OVA-challenged mice were suppressed by GPE and gallic acid treatments.

### 3.5. The Potential Antiallergic Mechanism of GPE

**Figure 9:**
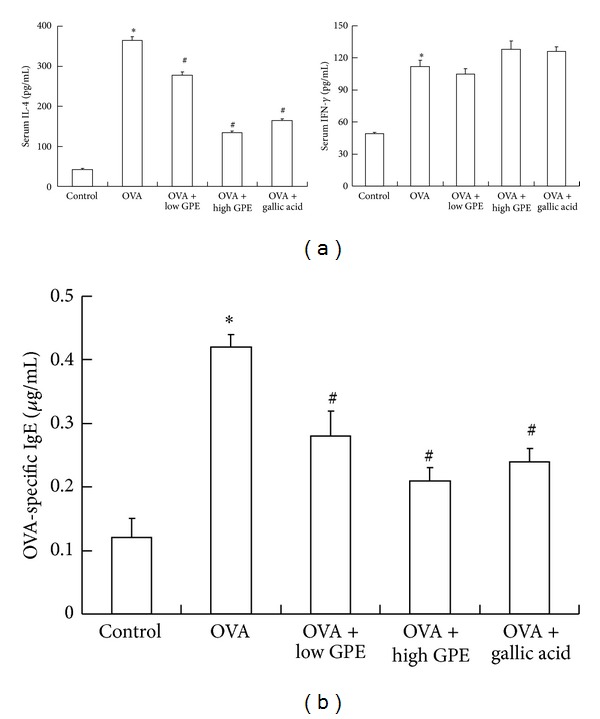
The antiallergic effects of GPE on serum (a) Th1 and Th2 cytokines and (b) OVA-specific IgE level. Data were shown as mean ± SEM (*n* = 12). *Significantly different from control group at *P* < 0.05. ^#^Significantly different from OVA-challenged group at *P* < 0.05.

Another allergic index, the serum OVA-specific IgE level, was investigated in this study. As shown in Figure 9, both GPE and gallic acid treatment markedly inhibited serum OVA-specific IgE levels of OVA-induced mice. With the exception of the BALF Th2 cytokines, the IL-4 and IFN-*γ* levels in the sera of the OVA-sensitized mice were determined on day 21 before the OVA challenge. Our results indicated that GPE and gallic acid significantly reduced serum IL-4 levels induced by OVA, but did not affect serum IFN-*γ* levels.

**Figure 10:**
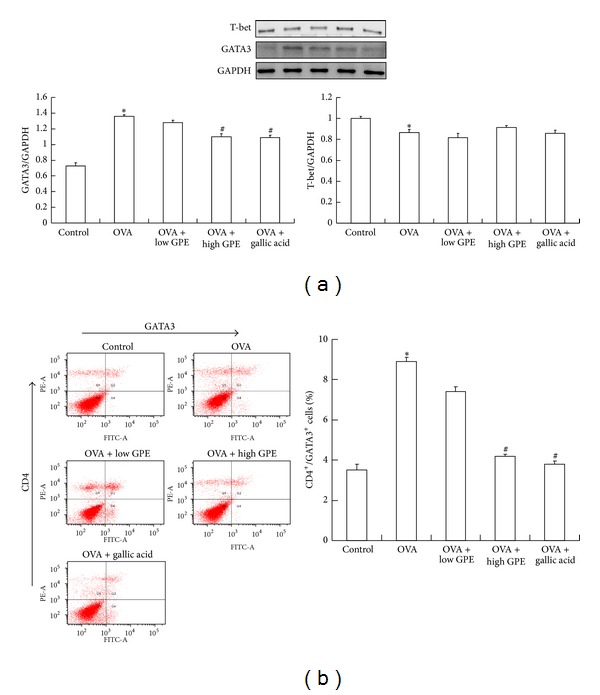
(a) The regulations of GPE on T-bet and GATA3 expressions of PBMCs by western blot. (b) The GATA3 expression of CD4 T cells by flow cytometry analysis. Cells in the lower right quadrant represented GATA3-positive cells, in the upper left quadrant represented CD4-positive cells, and in upper right quadrant represented CD4/GATA3-positive cells. The percentages of CD4/GATA3-positive cells were densotimetrically analyzed. Data were shown as mean ± SEM (*n* = 12). *Significantly different from control group at *P* < 0.05. ^#^Significantly different from OVA-challenged group at *P* < 0.05.

To better understand the antiallergic mechanism of GPE, we investigated Th1 and Th2 transcription factors T-bet and GATA3, respectively [26]. The level of GATA3 was markedly elevated by OVA induction, but T-bet levels in blood monocytes were not affected. Furthermore, treatment with GPE and gallic acid inhibited GATA3 expression in the blood monocytes of the OVA-induced mice (Figure 10(a)). Similarly, GPE and gallic acid also inhibited GATA3 expression in CD4 T cells induced by OVA treatment (Figure 10(b)). These findings demonstrate that GPE attenuates allergy and airway inflammation mediated by Th2 signal inhibition.

## 4. Discussion

Asthma is a chronic allergic disease characterized by airway inflammation and remodeling, bronchial hyperresponsiveness, variable airflow obstruction, and mucus hypersecretion [5, 6]. In this study, a mouse model of asthma was employed to assess the effect of GPE on airway inflammatory disease. Our results demonstrate that both GPE and its active compound gallic acid suppressed the allergy response and specifically IgE production to attenuate airway inflammation in the OVA-challenged mice. Our findings suggest that GPE or gallic acid has immunoregulatory effects on Th2 cells. We found that GPE significantly decreased the eosinophil, lymphocyte, and neutrophil levels in the BALF of allergic mice. Pathology analysis after treatment with GPE demonstrated reductions in allergic inflammatory infiltration, mucus secretion, and oxidative stress in the lung tissues. Moreover, Nrf2 activation (serine 40 phosphorylation) in the lungs of the OVA-challenged mice was increased by GPE or gallic acid treatments to increase the expression levels of GST and GCL, thereby attenuating lipid peroxidation.

Th2 lymphocytes play an important role in the initiation and progression of allergic diseases, including asthma, by releasing IL-4, IL-5, and IL-13 [5, 20]. IL-5 is a specific stimulator of eosinophil activation and is synthesized by Th2 lymphocytes, mast cells, and eosinophils [27]. In the pathogenesis of asthma, eosinophil migration into the lung is dependent on adhesion molecules [5, 6, 20]. Accumulating evidence has suggested that eosinophil interaction with epithelial cells via the adhesion molecule pathway is likely to be a key step for selective eosinophil recruitment [5, 6]. VCAM-1 and ICAM-1 are produced by epithelial cells in response to IL-4 and IL-13 and synergistically promote eosinophil recruitment into the lungs, along with IL-5 [20, 28]. The inhibition of VCAM-1 and ICAM-1 expression in the lungs of the OVA-challenged mice was suppressed by treatment with GPE and gallic acid (Figure 8).

Mucus is a viscoelastic gel that coats and lubricates the epithelium of mucosal surfaces. Low levels of mucus are produced in the normal lung, and mucus metaplasia with mucus hypersecretion is a characteristic feature of airways disorders such as asthma. Many studies have suggested that inflammatory cell infiltration and mucus hypersecretion are associated with the Th2 cytokines that regulate immune functions in mice. IL-4 was shown to play a key role in mucus production through the recruitment of Th2 cells into the lungs and the induction of inflammation [29]. The infiltration of inflammatory cells and mucus generation were both suppressed by GPE and gallic acid in OVA-challenged mice (Figures 4 and 5), and these effects were a result of the GPE-induced reduced Th2 cytokines levels (Figure 2).

Th2 cytokines induce inflammatory responses, such as airway infiltration, eosinophil activation, IgE production, and mucus secretion [29]. The elevated IgE levels observed in the early asthmatic response induce the degranulation of mast cells by the cross-linkage of allergen-specific IgE; this process is an important step in the development of asthmatic responses [30]. Clearly, GPE and gallic acid both inhibit OVA-specific IgE levels in the sera of OVA-sensitized mice, with this inhibitory effect being mediated by the GPE-induced inhibition of serum IL-4 (Figure 9).

IL-4 is synthesized by Th2 cells and plays an important role in allergic reactions. It is essential for IgE production, induces the differentiation of naive CD4 T cells into Th2 cells, and inhibits the functioning of Th1 cells. The cytokine IFN*γ* is produced primarily by natural killer and Th1 cells. It induces the differentiation of naive CD4 T cells into Th1 cells and inhibits the proliferation of Th2 cells [31]. GATA3 belongs to the GATA family of transcription factors, is an important regulator of T-cell development, and plays a role in endothelial cell biology. GATA3 promotes the secretion of IL-4, IL-5, and IL-13 from Th2 cells [32]. T-bet is expressed predominantly in Th1 cells and exhibits reciprocal inhibitory effects with GATA3 on T-cell differentiation [33]. Given that GPE did not affect the levels of Th1 cytokines (IFN-*γ*) in the sera of the OVA-sensitized mice but did attenuate IL-4 levels, we investigated the effect of GPE on Th2 signaling. We found that GPE and gallic acid markedly inhibited GATA3 levels (Figure 10).

Recently, several Chinese herbs have been reported to improve asthma and allergy through various mechanisms. For example, acacetin isolated from *Saussurea involucrata* was shown to inhibit Th2 cytokines [34]; bromelain and *Perillae fructus* were both found to attenuate asthma by restoring the Th1/Th2 imbalance in OVA-induced mice [35, 36]; and* Agaricus blazei* and* Panax ginseng* were shown to upregulate the Th1 response in intestinal epithelial cells to suppress OVA-sensitized allergy in mice [37, 38]. Moreover, the Cortex Mori Radicis extract was found to enhance CD4^+^CD25^+^Foxp3^+^ regulatory T cells and suppress Th2 cytokines, thereby improving asthma symptoms [39]. Recently, we found that rutin inhibits ionomycin/phorbol myristate acetate-induced Th2 signaling and GATA3 to attenuate allergy mediated by the activation of peroxisome proliferator activator receptor-gamma [26]. Goishi tea and green tea contain large amounts of gallic acid and have been shown to suppress IgE production and inhibit allergic asthma in humans [19, 40]. Similarly, GPE and gallic acid both inhibited IgE and Th2 signaling in OVA-sensitized mice in this study. With regard to other anti-allergic mechanisms, gallic acid has also been shown to inhibit the release of histamine and Th2 cytokines from mast cells and T cells [17, 18].

## 5. Conclusions

Our findings indicate that the administration of GPE and gallic acid both reduced the levels of serum and BALF IL-4, while it did not affect Th1 cytokine, suggesting that GPE and gallic acid modulate immune responses through inhibition of Th2 cytokine secretion. These results indicated that GPE has potentials for downregulating allergy by multimanners. In addition, GPE and gallic acid also reduce IgE level; however, the effects of GPE and gallic acid for B cell and dendritic cells remain unknown yet.
